# Supplementary material for: Web-based display of protein surface and pH-dependent properties for assessing the developability of biotherapeutics
Source: Sci Rep. 2019 Feb 13;9:1969. doi: 10.1038/s41598-018-36950-8 (PMC6374528; doi:10.1038/s41598-018-36950-8)
Supplement: Supplementary file 1 — Supplementary Information [file 41598_2018_36950_MOESM1_ESM.pdf]

Web-based display of protein surface and pH-dependent properties for assessing the developability of biotherapeutics

Max Hebditch[1] **and Jim Warwicker**[1*]

[1]School of Chemistry, Manchester Institute of Biotechnology, The University of Manchester, 131 Princess Street, Manchester, M1 7DN, UK
[*]jim.warwicker@manchester.ac.uk

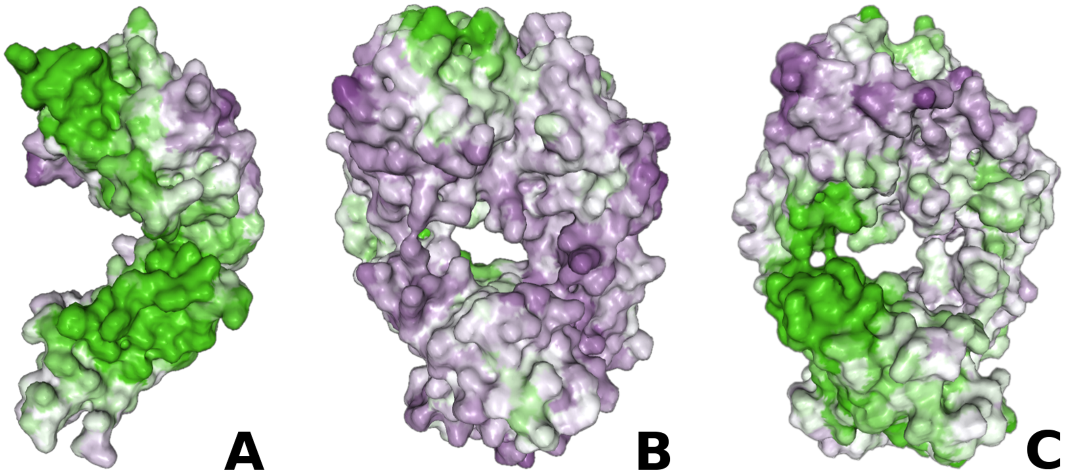

Figure S1. Protein-sol visualisations of the non-polar polar ratio of the three different Fabs with notable hydrophobic patch for each structural classification. In A, the heavy chain from the Fab fragment with the most hydrophobic patch in the Fab chain:chain interface, B, the Fab fragment with the most hydrophobic Fab:antigen interface patch and C, A Fab fragment with a large hydrophobic surface patch.

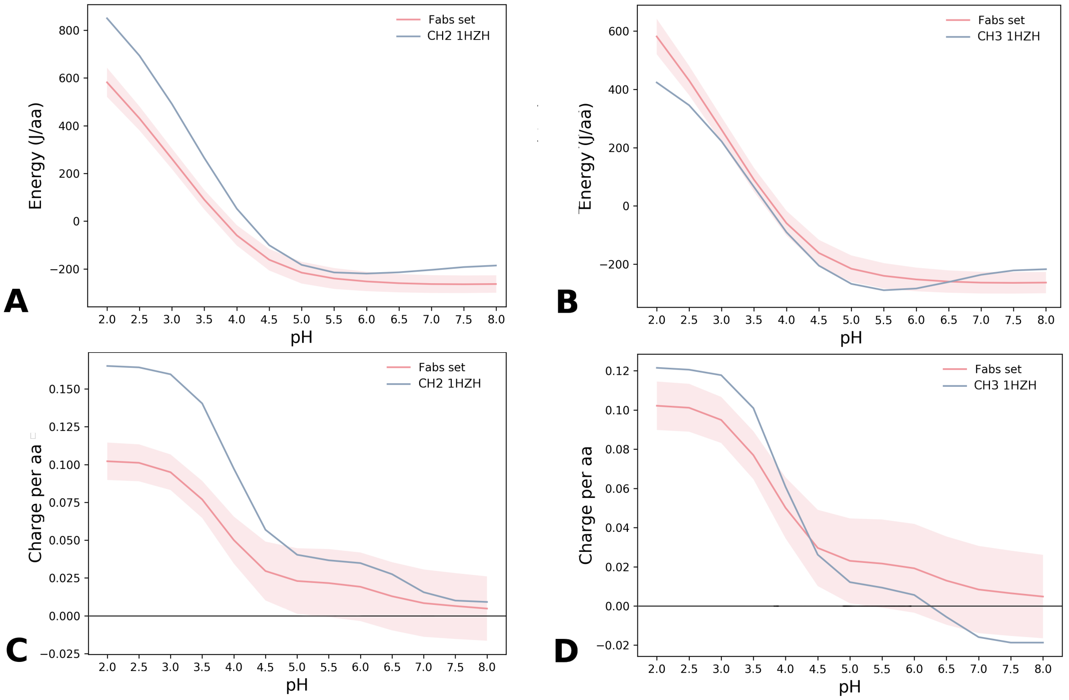

Figure S2. Representations of the stability and charge of the CH2 (A and C) and CH3 (B and D) domains from 1HZH for example purposes. Panels A and B compare the stability at 0M ionic strength across each pH value for CH2 and CH3, and panels C and D compare the charge at 0.3M ionic strength across each pH value for CH2 and CH3. The Fab dataset is also provided as a comparison, with the average for the entire represented by the red line, and the spread of the standard deviation in red.

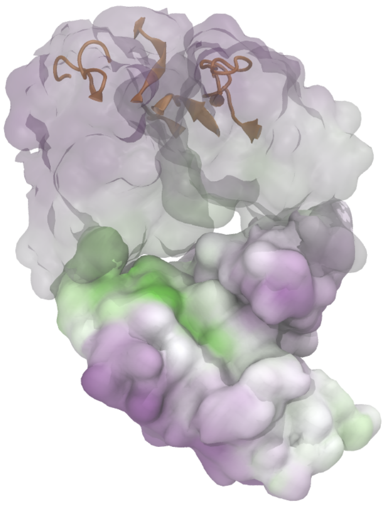

Figure S3. Example visualisation of the relatively high polarity at the Fab antigen interface in comparison to the rest of the Fab. Regions of high hydrophobicity are coloured green, low hydrophobicity coloured purple, and CDR regions highlighted in orange.

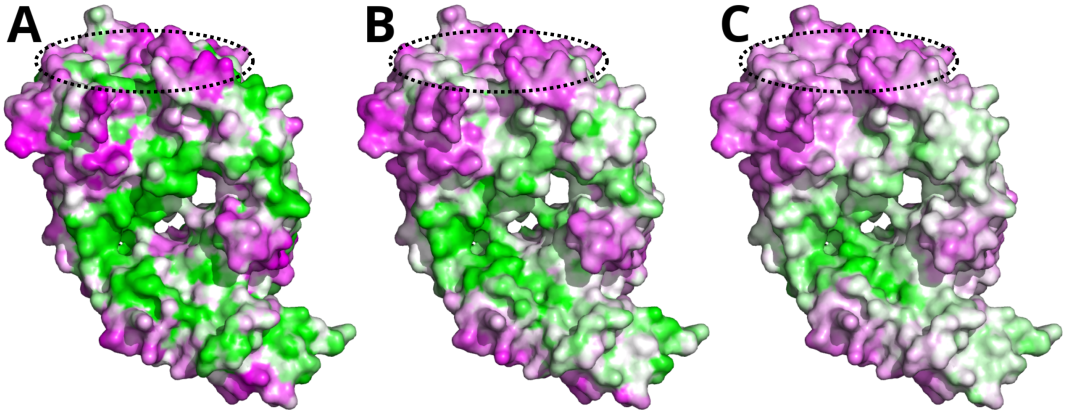

Figure S4. Comparison of varying patch radius in the protein-sol patch programme for calculating non-polar to polar ratio. In A, the radius is 7Å, in B, 10Å and in C, 13Å which is the default patch radius. In each figure, a region encompassing the CDRs is indicated. Visualisation was made using PyMOL.

	Fab surface	Monoenzyme surface	Fab chain:chain interface	Fab:antigen interface	Antigen surface
Fab surface		1.76E-121	0.00E+00	9.99E-115	1.96E-167
Monoenzyme surface	7.46E-01		0.00E+00	7.74E-228	3.28E-08
Fab chain:chain interface	5.23E-26	6.16E-14		0.00E+00	0.00E+00
Fab:antigen interface	2.83E-24	3.49E-17	3.39E-30		3.07E-240
Antigen surface	1.05E-04	1.56E-03	1.52E-17	1.50E-06	

Table S1. Statistical Comparison of Non-polar / polar (NPP) ratio distributions for different structural categorisations using the Mann-Whitney U test. In red, the distributions of the most hydrophobic patch for each protein, and in green the entire distribution of NPP values for each protein. Rather than true 0, values of 0.00E+00 lower are than the ability to precisely calculate.

ATOM	DESCRIPTION	UNITED-ATOM-RADIUS
O1	carbonyl-oxygen	1.40
O1O2H	carboxyl-oxygen	1.50
O2H	hydroxyl-oxygen	1.60
N3H	peptide-nitrogen	1.70
N3HH	terminal-nitrogen	1.80
N4HHH	terminal-nitrogen	2.00
N3H	aromatic-nitrogen	1.70
C3	bare-carbon	1.70
C4H	aliphatic-CH	2.00
C4HH	aliphatic-CH2	2.00
C4HHH	aliphatic-CH3	2.00
C3H	aromatic-CH-5-ring	1.85
C3H	aromatic-CH-6-ring	1.85
C3	bare-carbon-5/6-ring	1.70
S	sulphur	1.85
N3HH	arginine-NH2	1.80
N3H	arginine-NH	1.70

Table S2. United atom VDW (Å) radii in amino acids.

mainchain-peptide	N	-0.280
mainchain-peptide	H	0.280
mainchain-peptide	C	0.380
mainchain-peptide	O	-0.380
sidechain-arg	NH1	0.500
sidechain-arg	NH2	0.500
sidechain-asn	CG	0.380
sidechain-asn	OD1	-0.380
sidechain-asn	ND2	-0.830
sidechain-asn	HD21	0.415
sidechain-asn	HD22	0.415
sidechain-asp	CG	0.000
sidechain-asp	OD1	-0.500
sidechain-asp	OD2	-0.500
sidechain-gln	CD	0.380
sidechain-gln	OE1	-0.380
sidechain-gln	NE2	-0.830
sidechain-gln	HE21	0.415
sidechain-gln	HE22	0.415
sidechain-glu	CD	0.000
sidechain-glu	OE1	-0.500
sidechain-glu	OE2	-0.500
sidechain-his	ND1	0.500
sidechain-his	NE2	0.500
sidechain-lys	NZ	1.000
sidechain-ser	CB	0.150
sidechain-ser	OG	-0.548
sidechain-ser	HG	0.398
sidechain-thr	CB	0.150
sidechain-thr	OG1	-0.548
sidechain-thr	HG1	0.398
sidechain-trp	NE1	-0.050
sidechain-trp	HE1	0.190
sidechain-tyr	CZ	0.150
sidechain-tyr	OH	-0.548
sidechain-tyr	HH	0.398
mainchain-nt	NT	1.000
mainchain-ct	CT	0.000
mainchain-ct	OT1	-0.500
mainchain-ct	OT2	-0.500

Table S3. Amino acid atom charges (e) used in the current study, with histidine sidechain charge included at half the Table value.
